# Pathological diagnostic nomograms for predicting malignant histology and unfavorable pathology in patients with endophytic renal tumor

**DOI:** 10.3389/fonc.2022.964048

**Published:** 2022-09-21

**Authors:** Xinxi Deng, Xiaoqiang Liu, Bing Hu, Ming Jiang, Ke Zhu, Jianqiang Nie, Taobin Liu, Luyao Chen, Wen Deng, Bin Fu, Situ Xiong

**Affiliations:** ^1^Department of Urology, The First Affiliated Hospital of Nanchang University, Nanchang, China; ^2^Department of Urology, Jiu Jiang NO.1 People’s Hospital, Jiujiang, China; ^3^Jiangxi Institute of Urology, Nanchang, China

**Keywords:** endophytic renal tumor, pathological feature, malignant histology, unfavorable pathology, the R.E.N.A.L. Nephrometry Score, pathological diagnostic model, nomogram

## Abstract

**Purpose:**

To develop and validate nomograms for pre-treatment prediction of malignant histology (MH) and unfavorable pathology (UP) in patients with endophytic renal tumors (ERTs).

**Methods:**

We retrospectively reviewed the clinical information of 3245 patients with ERTs accepted surgical treatment in our center. Eventually, 333 eligible patients were included and randomly enrolled into training and testing sets in a ratio of 7:3. We performed univariable and multivariable logistic regression analyses to determine the independent risk factors of MH and UP in the training set and developed the pathological diagnostic models of MH and UP. The optimal model was used to construct a nomogram for MH and UP. The area under the receiver operating characteristics (ROC) curves (AUC), calibration curves and decision curve analyses (DCA) were used to evaluate the predictive performance of models.

**Results:**

Overall, 172 patients with MH and 50 patients with UP were enrolled in the training set; and 74 patients with MH and 21 patients with UP were enrolled in the validation set. Sex, neutrophil-to-lymphocyte ratio (NLR), R score, N score and R.E.N.A.L. score were the independent predictors of MH; and BMI, NLR, tumor size and R score were the independent predictors of UP. Single-variable and multiple-variable models were constructed based on these independent predictors. Among these predictive models, the malignant histology-risk nomogram consisted of sex, NLR, R score and N score and the unfavorable pathology-risk nomogram consisted of BMI, NLR and R score performed an optimal predictive performance, which reflected in the highest AUC (0.842 and 0.808, respectively), the favorable calibration curves and the best clinical net benefit. In addition, if demographic characteristics and laboratory tests were excluded from the nomograms, only the components of the R.E.N.A.L. Nephrometry Score system were included to predict MH and UP, the AUC decreased to 0.781 and 0.660, respectively (P=0.001 and 0.013, respectively).

**Conclusion:**

In our study, the pathological diagnostic models for predicting malignant and aggressive histological features for patients with ERTs showed outstanding predictive performance and convenience. The use of the models can greatly assist urologists in individualizing the management of their patients.

## Introduction

With the gradual increase in health awareness and the development of diagnostic imaging techniques, the frequency of accidental detection of small renal masses (SRMs,<4cm in diameter) is increasing in clinical work, accounting for approximately 50% of new renal cell carcinoma (RCC) diagnoses. Although most endophytic renal tumors (ERTs) are among them, they account for a very low percentage of SRMs due to their specific anatomical features and have been recognized as a surgical challenge.

ERTs were defined as tumors surrounded by normal renal parenchyma and attributed to 3 points of the E element in the R.E.N.A.L. Nephrometry Score (RENAL-NS) system (1, 2). These tumors are classified as complex renal tumors with higher R.E.N.A.L score because of the small size that does not protrude from the renal surface and the deep location near the renal collecting system. Partial nephrectomy (PN) has become the gold standard for the treatment of SRMs with better long-term benefits (3, 4). However, when the tumors are endophytic, the treatment options should be reappraised, as PN for them carries an increased surgical challenge and risk of perioperative complications (5, 6). Additionally, radical nephrectomy (RN) for ERTs simplifies surgical steps but with limited long-term benefits. It’s reported that up to 25% of surgically removed ERTs are benign (7), which challenges the necessity of surgery for ERTs once the potential benefits of surgical intervention are outweighed by the competing risks of mortality. Therefore, active surveillance (AS) has been recommended especially for patients who cannot tolerate surgery or are unwilling to undergo surgery by the National Comprehensive Cancer Network (NCCN) and the American Urological Association (AUA) (8, 9). Therefore, the pathological features of the tumor play a crucial role in determining the treatment choice for patients with ERTs.

Herein, we identified and quantified influence factors that increased the risk of malignant histology (MH) and unfavorable pathology (UP) for patients with ERTs. In addition, we first constructed pathological diagnostic models which combined demographic characteristics, laboratory tests and anatomical characteristics to predict histological features for ERTs and provided a pathological assessment tool.

## Materials and methods

### Patient cohort

**Figure 1:**
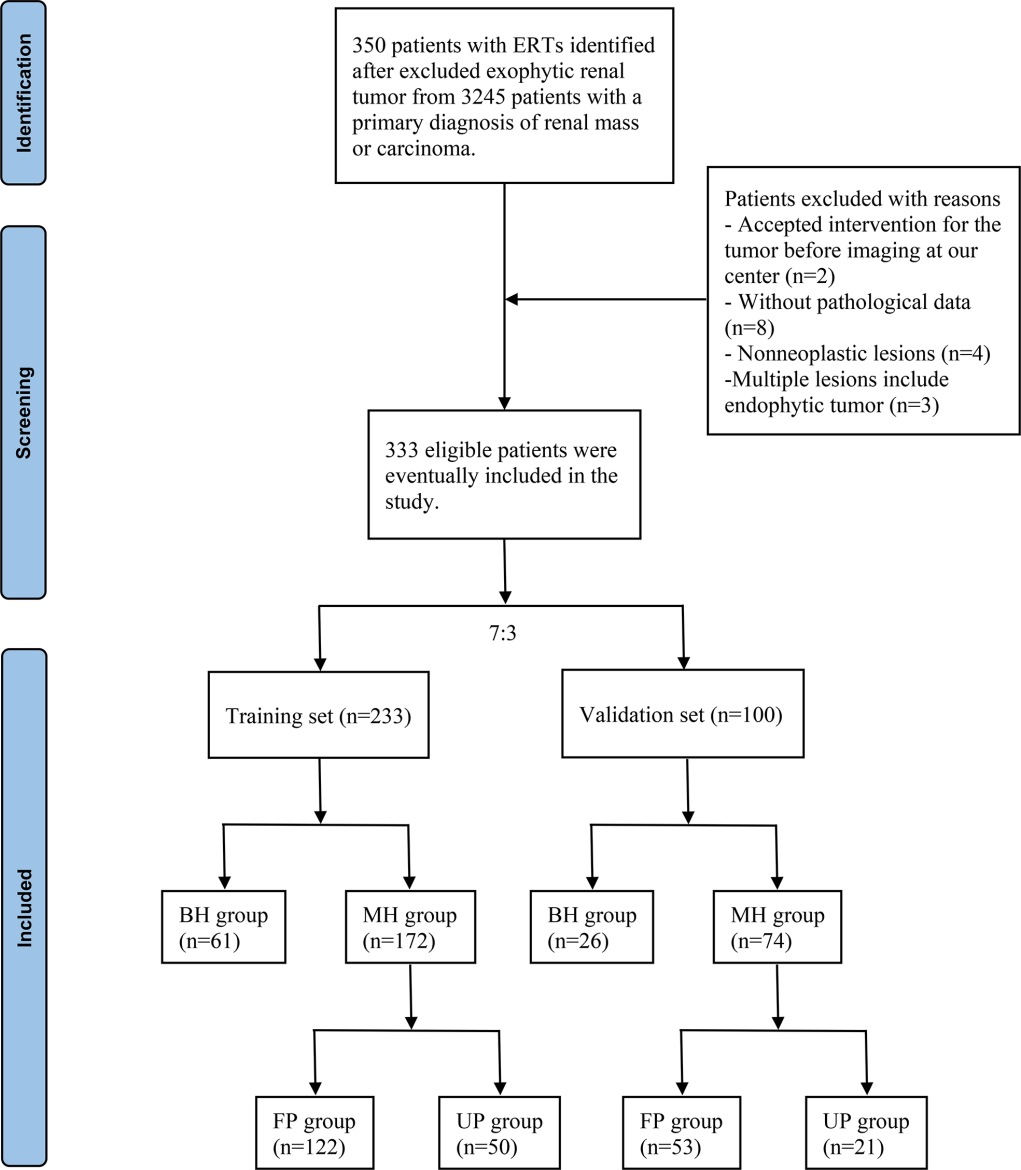
Flow chart of the included patients. ERTs, endophytic renal tumors; BH, benign histology; MH, malignant histology; FP, favorable pathology; UP, unfavorable pathology.

The radiographic database was used to search for cases of ERTs from 3245 patients who were initially diagnosed with renal mass or carcinoma between June 1, 2012, and June 1, 2022, in the First Affiliated Hospital of Nanchang University. Exclusion criteria were: (1) Patients with exophytic renal tumor; (2) Patients who had accepted intervention for the tumor before imaging examination in our center; (3) Patients who did not undergo surgery or percutaneous renal biopsy in the study center, i.e., those without pathological data; (4) The pathological diagnosis results were “pyelonephritis”, “renal tuberculosis”, “kidney disease” and other non-neoplastic lesions; (5) Multiple lesions include endophytic masses (n>3), such as multiple renal cysts and renal angiomyolipomas with endophytic lesions. The flow chart for screening patients with ERTs was shown in **Figure 1**.

### Research materials

Demographic characteristics, including age, sex, body mass index (BMI), diabetes mellitus and hypertension, and laboratory tests, including serum creatinine (Scr), hemoglobin (Hb), total cholesterol (TC), neutrophil to lymphocyte ratio (NLR), platelet to lymphocyte ratio (PLR), lymphocyte to monocyte ratio (LMR), albumin to globulin ratio (AGR) and prognostic nutrition index (PNI), were extracted from the prospectively managed clinical database. Tumor anatomical characteristics, including laterality, tumor size and components of the R.E.N.A.L.-NS system, were collected for all identified patients by reviewing the radiographic database. In the R.E.N.A.L.-NS system, a score of 1 to 3 was assigned to each of four key anatomical characteristics (R, E, N and L score) based on the size and location of tumors (**Supplement Table 1**). All cases scored 3 points in E score according to the definition. In addition, “h” indicates that the tumor was located in the renal hilar. The R.E.N.A.L. score of 4-6, 7-10, 10-12 were considered as low, moderate and high anatomical complexity, respectively. The radiographic data of all patients were independently reviewed and scored by Situ Xiong and Ming Jiang, who received professional training in image reading, and if necessary, the disputed scores were corrected by Luyao Chen, a senior doctor. All tumor specimens were reviewed by a single urological pathologist for pathological diagnoses. The pathological features consisted of histological subtype, pTNM stage and Fuhrman nuclear grade (I/II grade were classified into low grade and III/IV into high grade) according to the eighth edition of the American Joint Committee on Cancer (AJCC) cancer staging manual.

### Development, performance, and validation of the nomograms

Included patients were randomly divided into the training and validation sets in a ratio of 7:3. Then, patients were divided into the benign histology (BH) group and the malignant histology (MH) group based on histology types in the two sets, respectively. Those diagnosed with MH were further enrolled into the favorable pathology (FP) and unfavorable pathology (UP) groups based on pathological features. Cases were defined as UP following adverse histological features: pT3-4 RCC, high Fuhrman grade RCC, RCC with necrosis, RCC with lymphatic or vascular invasion, type II papillary RCC, and RCC with rhabdoid or sarcomatoid histology (10–12). All categorical variables compared using the Pearson χ2 test were presented in the form of numbers and percentages. As for continuous variables, the normally distributed variables using the Student t test presented as mean and standard deviation, and the non-normally distributed variables using the Wilcoxon rank sum test presented as median and interquartile range. We used binary univariable and multivariable logistic regression analyses to determine the independent risk factors of MH and UP in the training set. Then, these independent factors were used to construct single-variable and multiple-variable models to predict MH and UP. If there were multicollinearity between different variables in the multiple-variable model, we constructed different models by combining each of them with other clinically relevant and significant predictors. The receiver operating characteristic (ROC) curve and the area under the curve (AUC) were conducted to assess the predictive performance of different models in both the training and validation sets. Eventually, optimal models were used to develop the malignant histology-risk nomogram and the unfavorable pathology-risk nomogram based on the results of ROC analyses. Calibration curves were used to evaluate the calibration of the pathological diagnostic nomograms. Decision curve analyses (DCA) were used to evaluate the clinical net benefit, which can determine the clinical utility of the nomograms.

### Statistical analysis

All statistical analyses and graphs were performed using SPSS 24.0 software (SPSS, Chicago, IL, USA) and R software (version4.1.0). All tests were two-sided, and statistical significance was associated with a *P* < 0.05.

## Results

### Characteristics of the study cohort

After a retrospective chart review, the clinical data of 333 patients with ERTs who underwent PN and RN were used for analyses. Finally, 233 patients were enrolled in the training set, of which 61 and 172 were in the BH and MH groups, respectively, and 122 and 50 were in the FP and UP groups, respectively. The validation set included 100 patients with ERTs, including 26 in the BH group, 74 in the MH group, 53 in the FP group, and 21 in the UP group. **Tables 1** and **2** summarized the clinical characteristics of the groups in the training and validation sets. In the training set, sex (*P*<0.001), NLR (*P*<0.001), R score (*P*<0.001), N score (*P*<0.001), L score (*P*<0.001) and R.E.N.A.L. score (*P*<0.001) significantly differed between the BH and MH groups; and BMI (*P*<0.001), NLR (*P*<0.001), PLR (*P*=0.029) tumor size (*P*=0.001) and R score (*P*<0.001) significantly differed between the FP and UP groups. There was no statistical difference in other variables between groups (*P* > 0.05). In addition, no significant statistical difference was observed between the training and validation sets (*P* > 0.05).

**Table 1:** Demographic and clinical characteristics of the training and validation sets in BH vs. MH cohort.

	Training set (233)				Validation set (100)	*p* value
	Total	BH (61)	MH (172)	*p* value		
**Demographic characteristics**						
Age, years, mean (SD)	48.91 (14.00)	48.30 (10.45)	49.13 (15.09)	0.635	48.32 (13.88)	0.722
Sex (male), n (%)	135 (57.9)	22 (36.1)	113 (65.7)	**<0.001**	54 (54.0)	0.506
BMI, mean (SD)	23.50 (2.76)	23.27 (2.35)	23.58 (2.89)	0.443	23.04 (2.02)	0.093
Diabetes mellitus, n (%)	19 (8.2)	2 (3.3)	17 (9.9)	0.105	10 (10.0)	0.584
Hypertension, n (%)	38 (16.3)	7 (11.5)	31 (18.0)	0.234	14 (14.0)	0.595
**Laboratory tests**						
Scr, mg/dL, mean (SD)	0.88 (0.39)	0.84 (0.18)	0.89 (0.44)	0.349	0.87 (0.21)	0.882
Hb, g/dl, mean (SD)	131.55 (15.84)	132.56 (12.11)	131.19 (16.99)	0.498	129.64 (8.23)	0.153
TC, mmol/L, mean (SD)	4.44 (0.91)	4.42 (0.76)	4.45 (0.96)	0.797	4.34 (0.34)	0.141
NLR, mean (SD)	2.14 (1.12)	1.62 (0.82)	2.32 (1.15)	**<0.001**	2.20 (1.02)	0.628
PLR, mean (SD)	142.91 (58.31)	138.65 (46.59)	141.31 (57.00)	0.744	132.83 (39.11)	0.066
LMR, mean (SD)	4.62 (2.25)	4.94 (2.25)	4.51 (2.25)	0.200	4.73 (2.05)	0.668
AGR, mean (SD)	1.62 (0.26)	1.64 (0.22)	1.62 (0.27)	0.465	1.61 (0.16)	0.704
PNI, mean (SD)	50.20 (5.23)	50.25 (4.33)	50.18 (5.52)	0.921	49.99 (3.05)	0.652
**Anatomical features**						
Laterality (right), n (%)	111 (47.6)	29 (47.5)	82 (47.7)	0.986	51 (51.0)	0.574
Tumor size, cm, mean (SD)	3.19 (1.14)	3.00 (0.90)	3.26 (1.20)	0.079	3.29 (0.92)	0.393
R.E.N.A.L.-NS system						
R score, mean (SD)	1.33 (0.47)	1.08 (0.28)	1.41 (0.49)	**<0.001**	1.30 (0.46)	0.639
N score, mean (SD)	2.66 (0.70)	2.11 (0.92)	2.85 (0.47)	**<0.001**	2.66 (0.57)	0.990
L score, mean (SD)	2.35 (0.82)	2.02 (0.85)	2.47 (0.78)	**<0.001**	2.36 (0.77)	0.898
Hilar location, n (%)	64 (27.5)	13 (21.3)	51 (29.7)	0.210	26 (26.0)	0.782
RENAL score, mean (SD)				**<0.001**		0.358
4-6 (low complexity)	20 (8.6)	15 (24.6)	5 (2.9)		5 (5.0)	
7-9 (moderate complexity)	83 (35.6)	27 (44.3)	56 (32.6)		42 (42.0)	
10-12 (high complexity)	130 (55.8)	19 (31.1)	111 (64.5)		53 (53.0)	
**Pathologic characteristics**						
Malignant, n (%)	172 (73.8)				74 (74.00)	0.421
Clear cell RCC, n (%)		–	139 (80.8)	–	55 (74.3)	
Papillary RCC, n (%)		–	13 (7.6)	–	10 (13.5)	
Chromophobe RCC, n (%)		–	7 (4.1)	–	2 (2.7)	
Other, n (%)		–	13 (7.6)	–	7 (9.5)	
Benign, n (%)	61 (26.2)				26 (26.0)	0.636
Angiomyolipoma, n (%)		47 (77.1)	–		17 (65.4)	
Oncocytoma, n (%)		3 (4.9)	–		2 (7.7)	
Papillary adenoma, n (%)		4 (6.6)	–		3 (11.5)	
Other, n (%)		7 (11.5)	–		4 (15.4)	

BH, benign histology; MH, malignant histology; BMI, body mass index; Scr, serum creatinine; Hb, hemoglobin; TC, total cholesterol; NLR, neutrophil to lymphocyte ratio; PLR, platelet to lymphocyte ratio; LMR, lymphocyte to monocyte ratio; AGR, albumin to globulin ratio; PNI: prognostic nutrition index; R.E.N.A.L.-NS, RENAL- Nephrometry Score; RCC, renal cell carcinoma. Bolded numbers mean statistically different, i.e., *p* < 0.05.

**Table 2:** Demographic and clinical characteristics of the training and validation sets in FP vs. UP cohort.

	Training set				Validation set (74)	*p* value
	Total (172)	FP (122)	UP (50)	*p* value		
**Demographic characteristics**						
Age, years, mean (SD)	49.13 (15.09)	48.32 (14.50)	51.12 (16.42)	0.270	47.00 (13.71)	0.297
Sex (male), n (%)	113 (65.7)	81 (66.4)	32 (64.0)	0.764	47 (63.5)	0.742
BMI, mean (SD)	23.58 (2.89)	24.07 (2.71)	22.39 (3.01)	**<0.001**	22.92 (1.80)	0.068
Diabetes mellitus, n (%)	17 (9.9)	12 (9.8)	5 (10.0)	0.974	7 (9.5)	0.918
Hypertension, n (%)	31 (18.0)	20 (16.4)	11 (22.0)	0.385	9 (12.2)	0.253
**Laboratory tests**						
Scr, mg/dL, mean (SD)	0.89 (0.44)	0.87 (0.33)	0.93 (0.63)	0.448	0.87 (0.22)	0.673
Hb, g/dl, mean (SD)	131.19 (16.99)	131.60 (16.85)	130.18 (17.45)	0.620	129.86 (9.13)	0.431
TC, mmol/L, mean (SD)	4.45 (0.96)	4.45 (0.97)	4.46 (0.96)	0.937	4.35 (0.35)	0.259
NLR, mean (SD)	2.32 (1.15)	2.07 (0.98)	2.95 (1.30)	**<0.001**	2.38 (1.08)	0.737
PLR, mean (SD)	141.31 (57.00)	135.87 (53.79)	156.05 (62.28)	**0.029**	133.74 (42.24)	0.250
LMR, mean (SD)	4.51 (2.25)	4.67 (2.32)	4.11 (2.05)	0.139	4.62 (2.09)	0.725
AGR, mean (SD)	1.62 (0.27)	1.63 (0.28)	1.58 (0.25)	0.313	1.61 (0.18)	0.932
PNI, mean (SD)	50.18 (5.52)	50.34 (5.66)	49.78 (5.20)	0.540	49.97 (2.93)	0.701
**Anatomic features**						
Laterality (right), n (%)	82 (47.7)	56 (45.9)	26 (52.0)	0.467	38 (51.4)	0.597
Tumor size, cm, mean (SD)	3.39 (1.16)	3.20 (1.15)	3.83 (1.05)	**0.001**	3.42 (0.83)	0.765
R.E.N.A.L.-NS system						
R score, mean (SD)	1.41 (0.49)	1.32 (0.47)	1.64 (0.49)	**<0.001**	1.32 (0.47)	0.185
N score, mean (SD)	2.85 (0.47)	2.84 (0.50)	2.88 (0.39)	0.650	2.76 (0.52)	0.165
L score, mean (SD)	2.47 (0.78)	2.47 (0.77)	2.46 (0.81)	0.956	2.38 (0.74)	0.418
Hilar location, n (%)	51 (29.7)	34 (27.9)	17 (34.0)	0.424	22 (29.7)	0.990
RENAL score, mean (SD)				0.678		0.363
4-6 (low complexity)	5 (2.9)	4 (3.3)	1 (2.0)		3 (4.1)	
7-9 (moderate complexity)	56 (32.6)	42 (34.4)	14 (28.0)		30 (40.5)	
10-12 (high complexity)	111 (64.5)	76 (62.3)	35 (70.0)		41 (55.4)	
**Pathologic characteristics**						
Tumor histology				0.141		0.421
Clear cell RCC, n (%)	139 (80.8)	102 (83.6)	37 (74.0)		55 (74.3)	
Papillary RCC, n (%)	13 (7.6)	8 (6.6)	5 (10.0)		10 (13.5)	
Chromophobe RCC, n (%)	7 (4.1)	6 (4.9)	1 (2.0)		2 (2.7)	
Other, n (%)	13 (7.6)	6 (4.9)	7 (14.0)		7 (9.5)	
TNM stage						
T1a, n (%)	98 (57.0)	83 (68.0)	15 (30.0)	–	48 (64.9)	0.248
T1b, n (%)	45 (26.2)	39(32.0)	6 (14.2)	–	19 (25.7)	0.936
T3a, n (%)	22 (12.8)	–	22 (44.0)	–	6 (8.1)	0.289
T3b, n (%)	7 (4.1)	–	7 (14.0)	–	1 (1.4)	0.441
N1, n (%)	3 (1.7)	0	3 (6.0)	–	4 (5.4)	0.203
M1, n (%)	3 (1.7)	0	3 (6.0)	–	2 (2.7)	0.638
Tumor grade						
Fuhrman III-IV, n (%)	20 (13.8)	0	20 (52.6)	–	11 (16.9)	0.554

FP, favorable pathology; UP, unfavorable pathology; BMI, body mass index; Scr, serum creatinine; Hb, hemoglobin; TC, total cholesterol; NLR, neutrophil to lymphocyte ratio; PLR, platelet to lymphocyte ratio; LMR, lymphocyte to monocyte ratio; AGR, albumin to globulin ratio; PNI: prognostic nutrition index; R.E.N.A.L.-NS, RENAL- Nephrometry Score; RCC, renal cell carcinoma. Bolded numbers mean statistically different, i.e., p < 0.05.

### Univariable and multivariable analysis

**Figure 2:**
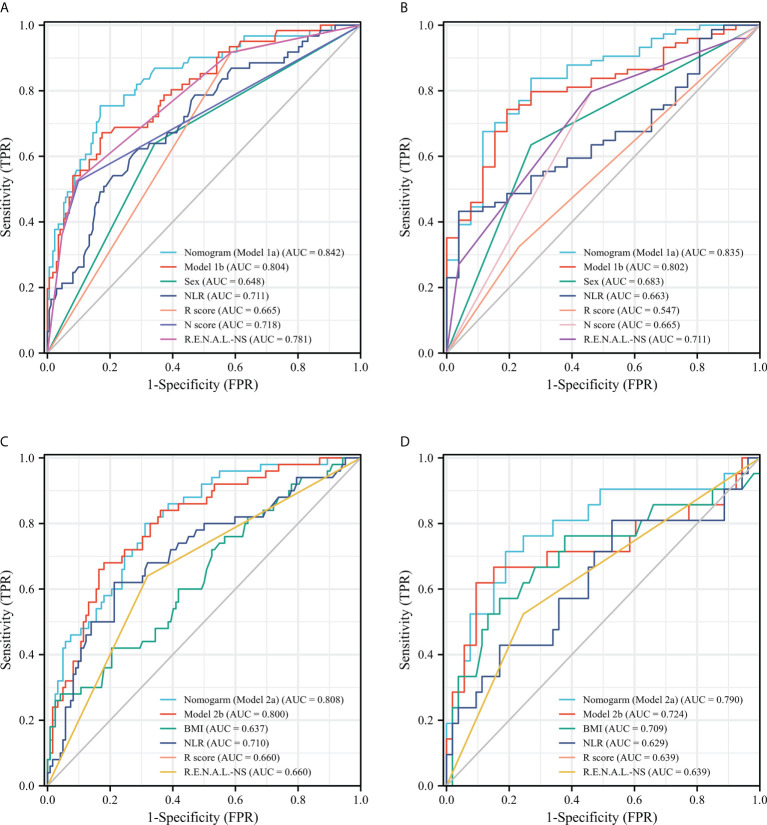
ROC curves of single-variable and multiple-variable models for evaluating the predictive performance of MH **(A, B)** and UP **(C, D)** in the training set **(A, C)** and the validation set **(B, D)**. ROC, receiver operating characteristic; MH, malignant histology; unfavorable pathology; NLR, neutrophil to lymphocyte ratio; R.E.N.A.L.-NS, RENAL- Nephrometry Score; BMI, body mass index.

**Figure 3:**
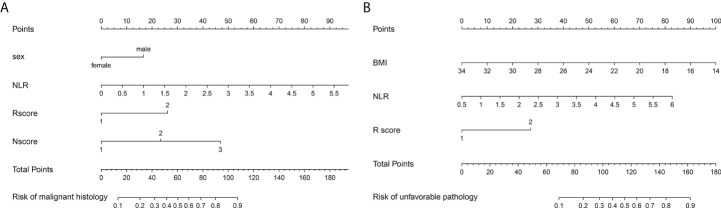
The malignant histology-risk nomogram **(A)** consisted of sex, NLR, R score and N score and the unfavorable pathology-risk nomogram **(B)** consisted of BMI, NLR and R score were developed to predict malignant and unfavorable pathology features for patients with ERTs. NLR, neutrophil to lymphocyte ratio; BMI, body mass index.

After univariable logistic regression analyses (**Table 3**), we observed that sex, NLR, R score, N score, L score, and R.E.N.A.L. score were the influential factors for ERTs to be MH (all *P*<0.05). BMI, NLR, PLR, tumor size and R score were the influential factors for ERTs to be UP (all *P*<0.05). To eliminate the collinearity within the R.E.N.A.L.-NS system, model 1a consisted of sex, NLR, R score, and N score, and model 1b consisted of sex, NLR, and R.E.N.A.L. score for MH were constructed based on multivariable analyses. In addition, based on multivariable analyses, model 2a consisted of BMI, NLR, and R score, and model 2b consisted of BMI, NLR, and tumor size for UP were constructed to eliminate the collinearity of tumor size and R score. The AUC of each model was calculated in both the training (**Figures 2A, C**) and validation sets (**Figures 2B, D**). Compared with model 1b, model 1a had a higher AUC in both the training and validation sets (0.842 vs. 0.804, *P*=0.039; 0.835 vs. 0.802, *P*=0.229, respectively). Model 2a achieved a higher AUC than model 2b in the training and validation sets (0.808 vs. 0.800, *P*=0.525; 0.790 vs. 0.724, *P*=0.141, respectively). In model 1a, male (OR, 2.22; 95% CI, 1.07-4.59; *P*=0.032), a higher NLR (OR, 2.18; 95% CI, 1.35-3.54; *P*=0.002), higher R score (OR, 3.32; 95% CI, 1.17-9.41; *P*=0.024) and higher N score (OR, 2.87; 95% CI, 1.66-4.96; *P*<0.001) were statistically significantly associated with an increased risk of MH (**Table 3**). In model 2a, a lager BMI (OR, 0.79; 95% CI, 0.68-0.91; *P*=0.001), higher NLR (OR, 2.17; 95% CI, 1.48-3.18; *P*<0.001) and higher R score (OR, 3.70; 95% CI, 1.70-8.07; *P*=0.001) increased the likelihood of MH (**Table 3**). Then we constructed the malignant histology-risk nomogram and the unfavorable pathology-risk nomogram according to models 1a (**Figure 3A**) and 2a (**Figure 3B**), respectively.

**Table 3:** Univariate and multiple logistic regressions evaluating the relationship of demographic and clinical characteristics with MH and UP.

Variables	Univariate Analysis		Multivariate Analysis			
	OR (95% CI)	*p* value	OR (95% CI)	*p* value	OR (95% CI)	*p* value
**BH vs. MH cohort**			**Model 1a**		**Model 1b**	
Age	1.00 (0.98-1.03)	0.687				
Sex			2.22 (1.07-4.59)	**0.032**	3.05 (1.52-6.09)	**0.002**
Female vs. Male	3.40 (1.85-6.25)	**<0.001**				
BMI	1.04 (0.94-1.16)	0.441				
Diabetes mellitus	3.24 (0.73-14.44)	0.124				
Hypertension	1.70 (0.71-4.08)	0.238				
Scr	1.61 (0.58-4.48)	0.358				
Hb	0.99 (0.98-1.01)	0.561				
TC	1.04 (0.75-1.43)	0.817				
NLR	2.33 (1.54-3.54)	**<0.001**	2.18 (1.35-3.54)	**0.002**	2.30 (1.44-3.66)	**<0.001**
PLR	1.00 (1.00-1.01)	0.591				
LMR	0.92 (0.82-1.04)	0.202				
AGR	0.65 (0.21-2.05)	0.462				
PNI	1.00 (0.94-1.06)	0.929				
Laterality						
Left vs. Right	1.01 (0.56-1.80)	0.986				
Tumor size	1.22 (0.95-1.58)	0.124				
R score	7.87 (3.00-20.64)	**<0.001**	3.32 (1.17-9.41)	**0.024**		
N score	4.06 (2.60-6.31)	**<0.001**	2.87 (1.66-4.96)	**<0.001**		
L score	1.90 (1.34-2.69)	**<0.001**	1.08 (0.66-1.75)	0.766		
Hilar location	1.56 (0.78-3.12)	0.212				
R.E.N.A.L. score						
4-6 vs.7-9	6.22 (2.05-18.91)	**0.001**			5.42 (1.63-18.05)	**0.006**
4-6 vs.10-12	17.53 (5.70-53.88)	**<0.001**			15.42 (4.64-51.18)	**<0.001**
**FP vs. UP cohort**			**Model 2a**		**Model 2b**	
Age	1.01 (0.99-1.04)	0.269				
Sex						
Female vs. Male	0.90 (0.45-1.79)	0.764				
BMI	0.80 (0.70-0.91)	**0.001**	0.79 (0.68-0.91)	**0.001**	0.77 (0.67-0.89)	**0.001**
Diabetes mellitus	1.02 (0.34-3.06)	0.974				
Hypertension	1.44 (0.63-3.28)	0.387				
Scr	1.31 (0.65-2.66)	0.455				
Hb	1.00 (0.98-1.01)	0.618				
TC	1.01 (0.72-1.43)	0.936				
NLR	1.94 (1.43-2.62)	**<0.001**	2.17 (1.48-3.18)	**<0.001**	2.09 (1.43-3.05)	**<0.001**
PLR	1.01 (1.00-1.01)	**0.033**	1.00 (0.99-1.00)	0.493	1.00 (0.99-1.00)	0.492
LMR	0.88 (0.75-1.04)	0.141				
AGR	0.53 (0.15-1.82)	0.312				
PNI	0.98 (0.92-1.04)	0.538				
Laterality						
Left vs. Right	1.28 (0.66-2.47)	0.468				
Tumor size	1.67 (1.22-2.29)	**0.002**			1.64 (1.15-2.33)	**0.006**
R score	3.78 (1.89-7.55)	**<0.001**	3.70 (1.70-8.07)	**0.001**		
N score	1.19 (0.56-2.53)	0.649				
L score	0.99 (0.65-1.51)	0.956				
Hilar location	1.33 (0.66-2.70)	0.425				
R.E.N.A.L. score						
4-6 vs.7-9	1.33 (0.14-12.95)	0.804				
4-6 vs.10-12	1.84 (0.20-17.09)	0.591				

BH, benign histology; MH, malignant histology; FP, favorable pathology; UP, unfavorable pathology; BMI, body mass index; Scr, serum creatinine; Hb, hemoglobin; TC, total cholesterol; NLR, neutrophil to lymphocyte ratio; PLR, platelet to lymphocyte ratio; LMR, lymphocyte to monocyte ratio; AGR, albumin to globulin ratio; PNI: prognostic nutrition index; R.E.N.A.L.-NS, RENAL- Nephrometry Score. Bolded numbers mean statistically different, i.e., p < 0.05.

### Performance of nomograms

**Figure 4:**
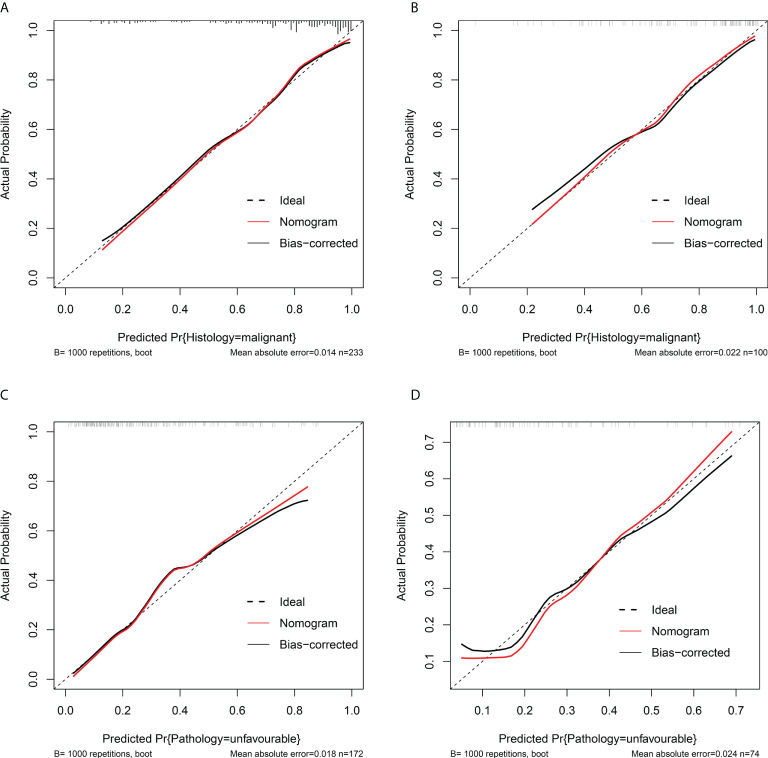
Calibration curves of the malignant histology-risk nomogram **(A, B)** and unfavorable pathology-risk nomogram **(B, D)** in the training set **(A, C)** and the validation set **(B, D)**. The 45° dotted diagonal line represents a perfect prediction, the red dashed line represents the predictive performance of the nomogram, together with a bias-corrected black solid line.

**Figure 5:**
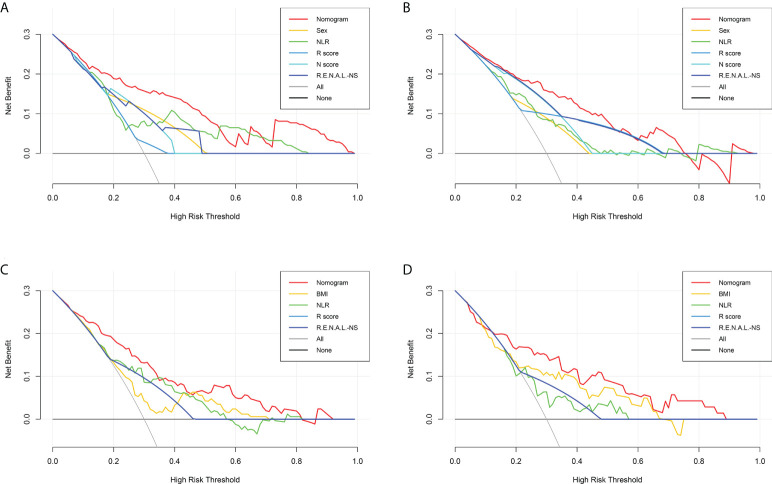
Decision curve analysis for the malignant histology-risk nomogram **(A, B)** and unfavorable pathology-risk nomogram **(C, D)** for evaluating the clinical utility in the training set **(A, C)** and validation set **(B, D)**. NLR, neutrophil to lymphocyte ratio; R.E.N.A.L.-NS, RENAL- Nephrometry Score; BMI, body mass index.

Among single- and multiple-variable models in identifying MH for ERTs, the malignant histology-risk nomogram (model 1a) had the highest AUC of 0.842 (95% CI, 0.782 to 0.901) in the training set (**Figure 2A**) and 0.835 (95% CI, 0.747 to 0.924) in the validation set (**Figure 2C**). If demographic characteristics and laboratory tests were excluded from the malignant histology-risk nomogram, only the components of R.E.N.A.L.-NS were included, the AUC decreased to 0.781 (*P*=0.001) in the training set and decreased to 0.711 (*P*=0.013) in the validation set (**Figures 2A, C**; **Table 4**). Among single- and multiple-variable models in identifying UP for ERTs, the unfavorable pathology-risk nomogram (model 2a) had the highest AUC of 0.808 (95% CI, 0.740 to 0.877) in the training set (**Figure 2B**) and 0.790 (95% CI, 0.661 to 0.918) in the validation set (**Figure 2D**). If demographic characteristics and laboratory tests were excluded from the malignant histology-risk nomogram, only the components of R.E.N.A.L.-NS were included, the AUC decreased to 0.660 (*P*<0.001) in the training set and decreased to 0.639 (*P*=0.036) in the validation set (**Figures 2B, D**; **Table 4**). The nomograms combining basic characteristics, laboratory tests and anatomical features have better predictive performance than the single-variable and R.E.N.A.L.-NS models in identifying MH and UP. The calibration curves of the malignant histology-risk nomogram and the unfavorable pathology-risk nomogram demonstrated good agreement between the actual observation and predicted probability in both sets (**Figures 4A–D**). The result of DCA curves demonstrated that using the nomograms to identify the pathologic features and make treatment decisions had a higher clinical benefit than either the “treat all” scheme or the “treat none” scheme. In addition, compared to single-variable and R.E.N.A.L.-NS models, both the malignant histology-risk and unfavorable pathology-risk nomograms performed better in the clinical decision (**Figures 5A–D**).

**Table 4:** Predictive performance outcomes of the nomogram and R.E.N.A.L.-NS.

Group	Nomogram				R.E.N.A.L.-NS model				*p* value
	ACC	SEN	SPE	AUC (95% CI)	ACC	SEN	SPE	AUC (95% CI)	
**BH vs. MH cohort**									
Training	0.80	0.77	0.81	0.85 (0.79-0.91)	0.80	0.53	0.91	0.80 (0.74-0.86)	0.005*
Validation	0.75	0.70	0.89	0.84 (0.75-0.92)	0.63	0.54	0.89	0.73 (0.63-0.84)	0.031*
**FP vs. UP cohort**									
Training	0.72	0.80	0.69	0.81 (0.74-0.88)	0.67	0.64	0.68	0.66 (0.58-0.74)	<0.001*
Validation	0.78	0.71	0.81	0.79 (0.66-0.92)	0.69	0.52	0.76	0.64 (0.52-0.76)	0.036*

ACC, accuracy; SEN, sensitivity; SPE, specificity; AUC, area under the curve; R.E.N.A.L.-NS, RENAL- Nephrometry Score.

*Delong test was used to compare the AUC of the nomogram and R.E.N.A.L.-NS. Bolded numbers mean statistically different, i.e., p < 0.05.

## Discussion

It is always known that optimal management of tumors should balance the potential benefit of intervention with the competing risks of adverse effects and mortality. PN is the preferred treatment for ERTs but with great surgical difficulty and perioperative complications due to the complex anatomy. RN for ERTs has a low risk of perioperative complications but is limited with long-term benefits. In addition, it has been reported that up to 25% of surgically removed ERTs are benign (7), which challenges the necessity of surgery for ERTs.Therefore, international guidelines such as the NCCN and AUA have recommended active surveillance (AS) as an alternative treatment for patients who cannot tolerate surgery or are unsuitable for surgery. Thus, the pathological features of ERTs play an important role in patient management. CT and MRI are the basis for the diagnosis of renal tumors but with moderate specificity (70-80%) and low sensitivity (20%) for the diagnosis of malignant tumors and cannot reliably distinguish subtypes of RCC (13). If we can accurately evaluate the pathological characteristics of ERT before the treatment determination, it will help to individualize the treatment of the tumor. Thus, we wanted to explore and validate a safe and reliable diagnostic prediction model to evaluate the biological behavior of ERTs.

In our study, the probability of surgically resected ERTs as BH and UP were 26.1% and 28.8%, respectively, which is in line with the reported in the contemporary literature (14–17). The outcomes of this study showed that some demographic characteristics, inflammatory indicators and anatomical features of patients with ERTs were related to pathological features.

Male was significantly more represented in MH than in BH (65.7% vs. 36.1%, *P*<0.001) and was an independent risk factor of MH (OR, 2.22; 95% CI, 1.07-4.59; *P*=0.032) in the training set. As is well known, the sex ratio for malignant renal tumors has always been 2:1, regardless of age, geographical location and ethnic background (18, 19), which was consistent with this study. The present studies have demonstrated that sex played an important role in evaluating localized renal mass pathology, and the male was always independently associated with malignancy (20–23).

In the immuno-oncology era, several studies have reported the “obesity paradox” phenomenon for patients with adverse RCC. In 2006, Parker etal. (24) found that overweight and obese patients were more likely to develop less-aggressive tumors than normal-weight patients. Tsivian etal. (25) reported that higher BMI was associated with a lower grade of RCC in clinically localized renal masses. In the same year, Bertrand etal. (26) analyzed the associations between obesity metrics and R.E.N.A.L.-NS, tumor grade and tumor stage in 99 patients who underwent surgery. They found that patients with low-grade Fuhrman tumors had higher BMI than those with high-grade tumors. Similar to the previously published series, BMI was negatively associated with the UP in our study (OR, 0.79; 95% CI, 0.68-0.91; *P*=0.001). In addition, the meta-analysis further confirmed that greater BMI significantly improved the prognosis of patients with RCC (27).

It has been established that elevated NLR is associated with malignant pathological findings in a variety of solid tumors (prostate, endometrial, adrenal, lung, and thyroid cancers) (28–32), including, of course, renal tumors (33, 34). In addition, studies have also confirmed the association of NLR with prognosis in RCC patients. Retrospective research on 2039 patients who underwent surgery for renal tumors by Viers et al. suggested that NLR was a preoperative marker of biologically aggressive RCC (34). Kim et al. found that an elevated preoperative NLR was associated with higher-grade Fuhrman and pT3a stage in patients with ≤7 cm renal tumors (35). A mate analysis evaluated the value of preoperative NLR in predicting the prognosis of surgically resectable urinary cancers and revealed that high preoperative NLR was associated with a worse prognosis in RCC (OS: HR=2.06, 95%CI: 1.54-2.76, *P*=0.131; CSS: HR=2.46, 95%CI: 1.46-4.16, *P*=0.178) (36). Our results showed that NLR was not only independently associated with MH (OR, 2.18; 95% CI, 1.35-3.54; P=0.002) but also with UP (OR, 2.23; 95% CI, 1.50-3.31; P<0.001) for patients with ERTs. These results suggest that NLR may be important markers of biological behavior and have predictive utility in the pre-treatment management of patients with ERTs.

After the R.E.N.A.L.-NS, a system to quantitate the salient anatomy of renal masses, was introduced in 2009 (36), it has become an increasingly used method to predict pathologic features for renal tumors (37–41). In our study, Logistic analyses demonstrated that ERTs with higher R score (OR, 3.32; 95% CI, 1.17-9.41; P=0.024) and N score (OR, 2.87; 95% CI, 1.66-4.96; P=0.021) were independently associated with MH and higher R score (OR, 3.74; 95% CI, 1.71-8.17; P=0.001) was independently associated with UP. Several studies have demonstrated that tumor size was significantly correlated with malignant and adverse pathological features (4, 21, 42, 43, 45, 46). A retrospective study that included 592 patients with renal tumors by Violette, P. et al. showed that tumor size was independently associated with a higher probability of benign disease (22). Also, a study by Thompson, R. H. et al. has shown that the risks of malignancy and high-grade tumors increase with tumor size (47). Cuijian Zhang etal. (46) reported that the tumor size was larger, and the grade and stage were prone to higher. In their result, Fuhrman Grade III occurred in 6.9% of renal tumors 2.1 to 4.0 cm in diameter and 22.3% of those of 4.1 to 7 cm in diameter. Therefore, they regarded 4 cm as a key point in the dramatic change in tumor aggressiveness. A retrospective study by Correa, A. F. et al. conducted on 334 men with SMRs showed that malignancy and high Fuhrman grade occur more frequently when tumors near the collecting system and renal sinus (48). Other authors reported similar suggestions (37, 45). The definite mechanisms of how the N score relates to malignant histology have not been elucidated. But they (48) put forward a hypothesis that proximity to the collecting system is a surrogate for tumor residence within the unique microenvironment of the inner renal medulla. This hostile environment promotes tumor survival and progression. However, no statistically significant difference was observed in the N score between the FP and UP groups in our study. Due to the deep location of ERTs and the fact that they do not protrude from the renal surface, most tumors are located close to the renal collecting system. Additionally, the mean N score of ERTs was 2.81 in our study, which further explains this difference from other studies. Therefore, the absence of statistically significant differences in N scores between the UP and FP groups can be explained. With anatomical features of the tumor identified as a predictor of pathological features, the R.E.N.A.L.-NS system may be a valuable risk assessment tool for ERTs.

It seems logical to counsel patients with ERTs on their risk of MH and UP based on demographic characteristics, preoperative peripheral blood-derived systemic inflammatory response markers, and tumor anatomical features. Hence, we constructed several predictive models based on those independent predictors of MH and UP. In both the training and validation sets, the malignant histology-risk nomogram consisted of sex, NLR, R score and N score and the unfavorable pathology-risk nomogram consisted of BMI, NLR and R score outperformed other single-variable models in evaluating malignant and unfavorable pathology, respectively. Due to the practicality and convenience of the R.E.N.A.L.-NS, it was widely used to predict the pathological features of renal tumors. However, in contrast, several authors believed that R.E.N.A.L.-NS could not accurately predict malignancy or aggressiveness (49–52). In the current study, we found that when only the components of R.E.N.A.L.-NS were considered to construct diagnostic prediction models, the predictive performance of models would significantly decrease not only for MH (AUC, 0.842 vs. 0.781 in the training set, *P*<0.001; AUC, 0.835 vs. 0.711 in the validation set, *P*=0.013) but also for UP (AUC, 0.808 vs. 0.660 in the training set, *P*<0.001; AUC, 0.790 vs. 0.639 in the validation set, *P*=0.036) compared with nomograms. These results revealed that the predictive efficacy of the models could only be optimized when multiple factors are combined.

The renal mass biopsy is the method for histopathological diagnosis at pretreatment with high accuracy. The median overall malignancy diagnostic rate was reported to be 92% ([IQR]: 80.6–96.8%) by a meta-analysis, with a sensitivity and specificity of 99.7% (95% CI, 81.5–100%) and 93.2% (95% CI, 83.3–99.8%), respectively (53). Additionally, the renal mass biopsy was also highly accurate in determining tumor tissue type and tumor grade (the two-tier Fuhrman grading system), with a concordance probability with surgical pathology of 90.3% and 86.5%, respectively (53). However, the clinical application of biopsy is limited due to the concerns about the risk of seeding tumor cells *via* the needle tract, although it was extremely low when coaxial needles were used (54). Additionally, considering that preoperative biopsy of renal masses was not yet routinely performed in China, we developed a malignant histology-risk and an unfavorable pathology-risk nomogram to quantify the likelihood of pre-treatment histological features of ERTs. The outcomes of our study could improve the tumor risk assessment and thus further guide the management of ERTs.

Nevertheless, our study is not devoid of limitations. First, our study was retrospective research based on a single central database, so this study was subject to selection bias. Second, anatomical characterization of tumors was based on the RENAL-NS system by two-dimensional cross-sectional imaging. As the difference in the experience and subjective judgment between the observers, there were variabilities in the assignment of scores. However, the nephrometry scores were scored independently by two doctors who received professional training in image reading, and the disputed scores were corrected by a senior doctor, which mitigates the limiting factor of the reliability of this study. Third, the sample size of this study was small due to the particularly low incidence of ERTs that account for about 10.8% of all renal tumors at our center. Furthermore, some patients did not receive surgical treatment due to the high risk, which further reduced the sample size. For now, the models were developed with the relatively large sample size we can achieve. But, as far as we know, this is the first study to construct pathological diagnostic models for patients with ERTs, which offer an alternative pathological assessment tool for pre-treatment management.

## Conclusion

In our study, the pathological diagnostic models for predicting malignant and aggressive histological features for patients with ERTs showed outstanding predictive performance and convenience. The use of the models can greatly assist urologists in individualizing the management of their patients. These data, although encouraging, still await large-sample multicenter validation before being applied to clinical practice.

## Data availability statement

The raw data supporting the conclusions of this article will be made available by the authors, without undue reservation.

## Ethics statement

The study was approved by the Ethics Committee of the First Affiliated Hospital of Nanchang University, Nanchang, Jiangxi, China. Written informed consent for participation was not required for this study in accordance with the national legislation and the institutional requirements. Written informed consent was not obtained from the individual(s) for the publication of any potentially identifiable images or data included in this article.

## Author contributions

Conception and design: SX and BF. Data collection: SX and MJ. Data analysis and interpretation: SX, XD, BH, KZ, JN, and TL. Manuscript writing: SX and XD. Manuscript revising: XD, XL and LC. Language polishing: XD. All authors listed have made a substantial, direct, and intellectual contribution to the work and proved it for publication.

## Funding

This study was supported by the National Natural Science Foundation of P.R. China (Grant Nos. 81960512), Jiangxi Provincial “Double Thousand Plan” Fund Project (Grant No. jxsq2019201027).

## Acknowledgments

The authors thank all the people who support this study.

## Conflict of interest

The authors declare that the research was conducted in the absence of any commercial or financial relationships that could be construed as a potential conflict of interest.

## Publisher’s note

All claims expressed in this article are solely those of the authors and do not necessarily represent those of their affiliated organizations, or those of the publisher, the editors and the reviewers. Any product that may be evaluated in this article, or claim that may be made by its manufacturer, is not guaranteed or endorsed by the publisher.
