# Supplementary material for: Pathological diagnostic nomograms for predicting malignant histology and unfavorable pathology in patients with endophytic renal tumor
Source: Front Oncol. 2022 Sep 21;12:964048. doi: 10.3389/fonc.2022.964048 (PMC9532530; doi:10.3389/fonc.2022.964048)
Supplement: Supplementary file 1 [file DataSheet_1.docx]

| Supplement TABLE 1 \| R.E.N.A.L. Nephrometry Score system | | | |
| --- | --- | --- | --- |
|  | 1 point | 2 points | 3points |
| R: maximal diameter (cm) | ≤4 | 4-7 | ≥7 |
| E: exophytic/endophytic properties | ≥50% | ＜50% | Entirely endophytic |
| N: nearness of the tumor to the collecting system or sinus（mm） | ≥7 | 4-7 | ≤4 |
| A: anterior / posterior | No points given. Mass assigned a descriptor of a, p, or x | | |
| L: location relative to the polar lines*  *h：renal hilus | Entirely above the upper or below the lower polar line | Lesion cross polar line | ＞50% of mass is across polar line (a) or mass crosses the axial renal midline (b) or mass is entirely between the polar lines (c) |

| Supplement Table 2. Univariate logistic regression analysis for identifying malignant histology and unfavorable pathology in ERTs | | | | | |
| --- | --- | --- | --- | --- | --- |
| Variables | BH vs. MH cohort | |  | FP vs. UP cohort | |
|  | Crude OR (95% CI) | *p* value |  | Adjusted OR (95% CI) | *p* value |
| Age | 1.00 (0.98-1.03) | 0.687 |  |  |  |
| Gender |  |  |  |  |  |
| Female (Ref.) vs. male | 3.40 (1.84-6.25) | **<0.001** |  | 2.23 (1.05-4.72) | **0.036** |
| BMI | 1.04 (0.94-1.16) | 0.441 |  |  |  |
| Diabetes mellitus | 3.24 (0.56-1.80) | 0.124 |  |  |  |
| Hypertension | 1.70 (0.73-14.44) | 0.238 |  |  |  |
| Scr | 1.61 (0.58-4.48) | 0.358 |  |  |  |
| Hb | 0.99 (0.98-1.01) | 0.561 |  |  |  |
| TC | 1.04 (0.75-1.43) | 0.817 |  |  |  |
| NLR | 2.33 (1.54-3.54) | **<0.001** |  | 2.17 (1.34-3.52) | **0.002** |
| PLR | 1.00 (1.00-1.01) | 0.325 |  |  |  |
| LMR | 0.92 (0.82-1.04) | 0.202 |  |  |  |
| AGR | 0.65 (0.21-2.05) | 0.462 |  |  |  |
| PNI | 1.00 (0.94-1.05) | 0.929 |  |  |  |
| Laterality |  |  |  |  |  |
| Left (Ref.) vs. Right | 1.01 (0.56-1.80) | 0.986 |  |  |  |
| Tumor size | 1.26 (0.97-1.64) | 0.089 |  |  |  |
| R score | 7.87 (3.00-20.64) | **<0.001** |  | 9.54 (2.38-38.19) | **0.001** |
| N score | 4.05 (2.60-6.31) | **<0.001** |  | 6.12 (2.50-14.99) | **<0.001** |
| L score | 1.13 (0.79-1.61) | 0.506 |  |  |  |
| Hilar location | 1.56 (0.78-3.12) | 0.212 |  |  |  |
| R.E.N.A.L. score | 1.72 (1.39-2.12) | **<0.001** |  | 0.3 (0.11-0.81) | **0.018** |
| Low (Ref.) vs. Moderate | 5.60 (1.83-17.12) | **0.003** |  | 4.49 (0.39-52.38) | 0.231 |
| Low (Ref.) vs. High | 16.36 (5.28-50.7) | **<0.001** |  | 20.10 (0.48-846.39) | 0.116 |
| BMI: body mass index; ACCI: age-adjusted Charlson's comorbidity index; ECOG PS: Eastern Cooperative Oncology Group Performance Status; ASA: American Society of Anesthesiologists; eGFR: estimated glomerular filtration rate; RENAL-NS: RENAL- Nephrometry Score; RN: radical nephrectomy; PN: partial nephrectomy | | | | | |

| Supplement Table 2. Univariate logistic regression analysis for identifying malignant histology and unfavorable pathology in ERTs | | | | | |
| --- | --- | --- | --- | --- | --- |
| Variables | BH vs. MH cohort | |  | FP vs. UP cohort | |
|  | Crude OR (95% CI) | *p* value |  | Adjusted OR (95% CI) | *p* value |
| Age | 1.00 (0.98-1.03) | 0.687 |  |  |  |
| Gender |  |  |  |  |  |
| Female (Ref.) |  |  |  |  |  |
| Male | 3.40 (1.84-6.25) | **<0.001** |  | 2.23 (1.05-4.72) | **0.036** |
| BMI | 1.04 (0.94-1.16) | 0.441 |  |  |  |
| Diabetes mellitus | 3.24 (0.56-1.80) | 0.124 |  |  |  |
| Hypertension | 1.70 (0.73-14.44) | 0.238 |  |  |  |
| Scr | 1.61 (0.58-4.48) | 0.358 |  |  |  |
| Hb | 0.99 (0.98-1.01) | 0.561 |  |  |  |
| TC | 1.04 (0.75-1.43) | 0.817 |  |  |  |
| NLR | 2.33 (1.54-3.54) | **<0.001** |  | 2.17 (1.34-3.52) | **0.002** |
| PLR | 1.00 (1.00-1.01) | 0.325 |  |  |  |
| LMR | 0.92 (0.82-1.04) | 0.202 |  |  |  |
| AGR | 0.65 (0.21-2.05) | 0.462 |  |  |  |
| PNI | 1.00 (0.94-1.05) | 0.929 |  |  |  |
| Laterality |  |  |  |  |  |
| Left (Ref.) |  |  |  |  |  |
| Right | 1.01 (0.56-1.80) | 0.986 |  |  |  |
| Tumor size | 1.26 (0.97-1.64) | 0.089 |  |  |  |
| R score | 7.87 (3.00-20.64) | **<0.001** |  | 9.54 (2.38-38.19) | **0.001** |
| N score | 4.05 (2.60-6.31) | **<0.001** |  | 6.12 (2.50-14.99) | **<0.001** |
| L score | 1.13 (0.79-1.61) | 0.506 |  |  |  |
| Hilar location | 1.56 (0.78-3.12) | 0.212 |  |  |  |
| R.E.N.A.L. score | 1.72 (1.39-2.12) | **<0.001** |  | 0.3 (0.11-0.81) | **0.018** |
| 4-6 (Ref.) vs. |  |  |  |  |  |
| 7-9 | 5.60 (1.83-17.12) | **0.003** |  | 4.49 (0.39-52.38) | 0.231 |
| 10-12 | 16.36 (5.28-50.7) | **<0.001** |  | 20.10 (0.48-846.39) | 0.116 |
| BMI: body mass index; ACCI: age-adjusted Charlson's comorbidity index; ECOG PS: Eastern Cooperative Oncology Group Performance Status; ASA: American Society of Anesthesiologists; eGFR: estimated glomerular filtration rate; RENAL-NS: RENAL- Nephrometry Score; RN: radical nephrectomy; PN: partial nephrectomy | | | | | |

| Table 3. Univariate and multiple logistic regressions evaluating the relationship of demographic and clinical characteristics with malignant histology. | | | | | | | | |  |
| --- | --- | --- | --- | --- | --- | --- | --- | --- | --- |
| Variables | Univariate Analysis | |  | Multivariate Analysis for model 1a | |  | Multivariate Analysis for model 1b | | |
|  | Crude OR (95% CI) | *p* value |  | Crude OR (95% CI) | *p* value |  | Crude OR (95% CI) | *p* value | |
| Age | 20.10 (0.48-846.39) | **<0.001** |  | 20.10 (0.48-846.39) | **<0.001** |  | 20.10 (0.48-846.39) | **<0.001** | |
| Sex |  |  |  |  |  |  |  |  | |
| Female vs. Male |  |  |  |  |  |  |  |  | |
| BMI |  |  |  |  |  |  |  |  | |
| Diabetes mellitus |  |  |  |  |  |  |  |  | |
| Hypertension |  |  |  |  |  |  |  |  | |
| Scr |  |  |  |  |  |  |  |  | |
| Hb |  |  |  |  |  |  |  |  | |
| TC |  |  |  |  |  |  |  |  | |
| NLR |  |  |  |  |  |  |  |  | |
| PLR |  |  |  |  |  |  |  |  | |
| LMR |  |  |  |  |  |  |  |  | |
| AGR |  |  |  |  |  |  |  |  | |
| PNI |  |  |  |  |  |  |  |  | |
| Laterality |  |  |  |  |  |  |  |  | |
| Left vs. Right |  |  |  |  |  |  |  |  | |
| Tumor size |  |  |  |  |  |  |  |  | |
| R score |  |  |  |  |  |  |  |  | |
| N score |  |  |  |  |  |  |  |  | |
| L score |  |  |  |  |  |  |  |  | |
| Hilar location |  |  |  |  |  |  |  |  | |
| R.E.N.A.L. score |  |  |  |  |  |  |  |  | |
| 4-6 vs.7-9 |  |  |  |  |  |  |  |  | |
| 4-6 vs.10-12 |  |  |  |  |  |  |  |  | |
| Age |  |  |  |  |  |  |  |  | |
| Sex |  |  |  |  |  |  |  |  | |
| Female vs. Male |  |  |  |  |  |  |  |  | |
| BMI |  |  |  |  |  |  |  |  | |
| Diabetes mellitus |  |  |  |  |  |  |  |  | |
| Hypertension |  |  |  |  |  |  |  |  | |
| Scr |  |  |  |  |  |  |  |  | |
| Hb |  |  |  |  |  |  |  |  | |
| TC |  |  |  |  |  |  |  |  | |
| NLR |  |  |  |  |  |  |  |  | |
| PLR |  |  |  |  |  |  |  |  | |
| LMR |  |  |  |  |  |  |  |  | |
| AGR |  |  |  |  |  |  |  |  | |
| PNI |  |  |  |  |  |  |  |  | |
| Laterality |  |  |  |  |  |  |  |  | |
| Left vs. Right |  |  |  |  |  |  |  |  | |
| Tumor size |  |  |  |  |  |  |  |  | |
| R score |  |  |  |  |  |  |  |  | |
| N score |  |  |  |  |  |  |  |  | |
| L score |  |  |  |  |  |  |  |  | |
| Hilar location |  |  |  |  |  |  |  |  | |
| R.E.N.A.L. score |  |  |  |  |  |  |  |  | |
| 4-6 vs.7-9 |  |  |  |  |  |  |  |  | |
| 4-6 vs.10-12 |  |  |  |  |  |  |  |  | |

| Table 3. Univariate and multiple logistic regressions evaluating the relationship of demographic and clinical characteristics with malignant histology. | | | | | | | | |
| --- | --- | --- | --- | --- | --- | --- | --- | --- |
| Variables | Univariate Analysis | |  | Multivariate Analysis for model 1a | |  | Multivariate Analysis for model 1b | |
|  |  |  |  |  |  |  |  |  |
| Age | 20.10 (0.48-846.39) | **<0.001** |  | 20.10 (0.48-846.39) | **<0.001** |  | 20.10 (0.48-846.39) | **<0.001** |
| Gender |  |  |  |  |  |  |  |  |
| Female |  |  |  |  |  |  |  |  |
| Male |  |  |  |  |  |  |  |  |
| BMI |  |  |  |  |  |  |  |  |
| Diabetes mellitus |  |  |  |  |  |  |  |  |
| Hypertension |  |  |  |  |  |  |  |  |
| Scr |  |  |  |  |  |  |  |  |
| Hb |  |  |  |  |  |  |  |  |
| TC |  |  |  |  |  |  |  |  |
| NLR |  |  |  |  |  |  |  |  |
| PLR |  |  |  |  |  |  |  |  |
| LMR |  |  |  |  |  |  |  |  |
| AGR |  |  |  |  |  |  |  |  |
| PNI |  |  |  |  |  |  |  |  |
| Laterality |  |  |  |  |  |  |  |  |
| Left (Ref.) |  |  |  |  |  |  |  |  |
| Right |  |  |  |  |  |  |  |  |
| Tumor size |  |  |  |  |  |  |  |  |
| R score |  |  |  |  |  |  |  |  |
| N score |  |  |  |  |  |  |  |  |
| L score |  |  |  |  |  |  |  |  |
| Hilar location |  |  |  |  |  |  |  |  |
| R.E.N.A.L. score |  |  |  |  |  |  |  |  |
| 4-6 (Ref.) |  |  |  |  |  |  |  |  |
| 7-9 |  |  |  |  |  |  |  |  |
| 10-12 |  |  |  |  |  |  |  |  |

| Table 4. Univariate and multiple logistic regressions evaluating the relationship of demographic and clinical characteristics with unfavorable pathology. | | | | | | | | |
| --- | --- | --- | --- | --- | --- | --- | --- | --- |
| Variables | Univariate Analysis | |  | Multivariate Analysis for model 2a | |  | Multivariate Analysis for model 2b | |
|  |  |  |  |  |  |  |  |  |
| Age | 20.10 (0.48-846.39) | **<0.001** |  | 20.10 (0.48-846.39) | **<0.001** |  | 20.10 (0.48-846.39) | **<0.001** |
| Gender |  |  |  |  |  |  |  |  |
| Female |  |  |  |  |  |  |  |  |
| Male |  |  |  |  |  |  |  |  |
| BMI |  |  |  |  |  |  |  |  |
| Diabetes mellitus |  |  |  |  |  |  |  |  |
| Hypertension |  |  |  |  |  |  |  |  |
| Scr |  |  |  |  |  |  |  |  |
| Hb |  |  |  |  |  |  |  |  |
| TC |  |  |  |  |  |  |  |  |
| NLR |  |  |  |  |  |  |  |  |
| PLR |  |  |  |  |  |  |  |  |
| LMR |  |  |  |  |  |  |  |  |
| AGR |  |  |  |  |  |  |  |  |
| PNI |  |  |  |  |  |  |  |  |
| Laterality |  |  |  |  |  |  |  |  |
| Left (Ref.) |  |  |  |  |  |  |  |  |
| Right |  |  |  |  |  |  |  |  |
| Tumor size |  |  |  |  |  |  |  |  |
| R score |  |  |  |  |  |  |  |  |
| N score |  |  |  |  |  |  |  |  |
| L score |  |  |  |  |  |  |  |  |
| Hilar location |  |  |  |  |  |  |  |  |
| R.E.N.A.L. score |  |  |  |  |  |  |  |  |
| 4-6 (Ref.) |  |  |  |  |  |  |  |  |
| 7-9 |  |  |  |  |  |  |  |  |
| 10-12 |  |  |  |  |  |  |  |  |
